# Impact of fracture reduction quality on clinical outcomes in hip arthroplasty for intertrochanteric fractures based on a novel radiographic evaluation system: a retrospective study

**DOI:** 10.3389/fmed.2025.1637763

**Published:** 2025-08-14

**Authors:** Binquan Zhang, Jia Huo, Huijie Li

**Affiliations:** Department of Orthopedic Surgery, Hebei Medical University Third Hospital, Shijiazhuang, Hebei, China

**Keywords:** reduction, fracture, hip, arthroplasty, radiographic

## Abstract

**Background:**

The impact of fracture reduction quality on clinical outcomes in hip arthroplasty for intertrochanteric fractures remains insufficiently characterized. This study aimed to establish a standardized postoperative radiographic evaluation system for reduction quality and assess its correlation with postoperative function and complications.

**Methods:**

A retrospective cohort study included 237 patients undergoing hip arthroplasty for intertrochanteric fractures (2012–2024). Reduction quality was classified as optimal, acceptable, or poor based on four criteria: (1) greater trochanter alignment, (2) lesser trochanter reduction, (3) femoral stem stability, and (4) postoperative femoral anteversion (optimal: 13 ± 3°; acceptable: 6–10° or 16–20°; poor: <6° or >20°). Outcomes included Harris Hip Scores, Engh's scores, delayed healing, and complications. Statistical analyses were adjusted for AO/OTA fracture classification.

**Results:**

Optimal reduction (Grade A, *n* = 107) correlated with superior Harris Hip Scores (92.57 ± 4.27 vs. 82.46 ± 7.05, *P* < 0.001), lower delayed healing (3.74% vs. 14.29%, *P* = 0.031), and reduced abductor weakness (1.87% vs. 14.29%, *P* = 0.014). Acceptable reductions (Grade B, *n* = 74) showed intermediate outcomes. Poor reductions (Grade C, *n* = 56) exhibited the highest complication rates. Engh's scores were significantly higher in Grade A (97.20% vs. 73.21%, *P* = 0.002). Dislocation and heterotopic ossification rates did not differ significantly (*P* > 0.05).

**Conclusion:**

This study introduced and validated a standardized radiographic evaluation system to assess reduction quality in arthroplasty for intertrochanteric fractures, emphasizing the prognostic importance of anatomic trochanteric alignment and cortical continuity. High-quality reduction is critical for optimizing functional recovery and minimizing complications in arthroplasty for intertrochanteric fractures. Future research should explore long-term outcomes and advanced fixation techniques to enhance reduction precision.

## Introduction

Osteoporotic hip fractures, prevalent in aging populations, are associated with high mortality and socioeconomic burdens (1–4). Intertrochanteric fractures are about half as common as osteoporotic hip fractures (5, 6). Although internal fixation is the current treatment of choice for intertrochanteric fractures, a risk of failure persists, particularly in cases of unstable fracture or severe osteoporosis (7–10). Furthermore, patients with pre-existing hip pathologies, such as hip osteoarthritis or osteonecrosis of the femoral head, may face an increased likelihood of requiring subsequent surgical intervention following internal fixation for intertrochanteric femoral fractures (11).

Numerous studies have compared treatment approaches for intertrochanteric fractures. Intramedullary fixation, exemplified by the Gamma nail and Intertan double nail system, and extramedullary fixation using dynamic hip screws, demonstrated advantages over arthroplasty. These fixation techniques require shorter operative times, reduce intraoperative blood loss, decrease transfusion requirements, and preserve the native hip joint, ultimately yielding improved patient outcomes (6, 12–14). However, in certain situations such as unstable fractures, severe osteoporosis, or hip diseases such as femoral head necrosis and hip osteoarthritis, the failure rate of internal fixation devices will significantly increase (15, 16). Such failure carries catastrophic consequences, potentially resulting in severe hip joint function decline and complications including fixation loosening, cut-out, nonunion, and coxa vara subsequent to treatment (17–21). Therefore, increasing studies have shown that hip arthroplasty is superior to internal fixation in cases of unstable intertrochanteric fractures, or hip joint diseases such as severe osteoporosis, femoral head necrosis, and hip osteoarthritis (6, 22–26). Therefore, in select cases of intertrochanteric fractures, hip arthroplasty can be considered as the primary treatment strategy to avoid reoperation and enhance patient outcomes (27, 28).

The reduction of intertrochanteric fractures during arthroplasty procedures is a critical aspect of surgical technique. Disruption in the anatomical reduction of the greater trochanter, particularly in cases of comminuted fractures, can lead to compromised abductor muscle function. This dysfunction manifests clinically as pain and the characteristic Trendelenburg gait pattern, significantly affecting patients' quality of life (16, 21, 29). Recognizing this, surgeons often prioritize stable fixation of the greater trochanter fragment during hip arthroplasty for intertrochanteric fractures (30). Patients with lesser trochanter fractures often experienced prolonged impairment in hip flexion, as indicated by reduced iliopsoas muscle function on the Ludloff's test. This deficit may lead to difficulties in activities requiring hip flexion, such as standing from a seated position or ascending stairs (31). Although overall hip function scores may not show significant differences, the presence of a displaced lesser trochanter was associated with increased fatty infiltration of the iliopsoas muscle, which could contribute to long-term functional decline (32). However, no studies have investigated how to assess reduction quality during arthroplasty procedures for intertrochanteric fractures or its impact on hip function and postoperative complications.

Against this background, this retrospective cohort study pursued two primary objectives. First, it endeavored to establish a robust radiographic assessment criterion to assess reduction quality based on postoperative radiographs. Second, it sought to determine the necessity of achieving stable fracture reduction quality during hip arthroplasty for the treatment of intertrochanteric fractures. By analyzing the relationship between fracture reduction quality and clinical outcomes, this study aimed to optimize surgical techniques and improve prognoses for this challenging patient population.

## Materials and methods

### Study design and patient selection

Of 275 patients diagnosed with intertrochanteric fractures and treated with arthroplasty between March 2012 and January 2024, 237 patients were followed-up for at least 1 year, with a sufficient number of radiographs taken with an image intensifier during surgery, and were included in this retrospective research. Indications for hip arthroplasty included unstable fractures (AO/OTA 31–A2.3, A3.1–A3.3) with posteromedial comminution, pre-existing ipsilateral hip osteoarthritis (Kellgren-Lawrence grade ≥3), femoral head avascular necrosis, or severe osteoporosis (*T*-score ≤ −3.0) (22–25). Exclusion criteria included: (1) Incomplete imaging data; (2) Under 55 years old or over 90 years old; (3) Follow up time <1 year; (4) Postoperative periprosthetic joint infection (patients with functional impairments caused by other postoperative complications were excluded); (5) Pathological fracture; (6) Complicating other fracture; (7) Uncompleted clinical records; (8) Other accompanying diseases, such as rheumatoid arthritis or dysplasia of the hip joint; (9) The greater trochanter of the femur has been removed during the operation.

### Surgical procedure and postoperative management

All patients suffered fractures due to low-energy injuries caused by falling while walking, falling from a height of <2 m, or falling while riding a bicycle. According to the AO/OTA classification, patients were classified based on pelvic anterior-posterior radiographs before surgery. All patients underwent posterior approach incisions for hip arthroplasty, and received fracture reduction and internal fixation treatment during the operation according to the situation. The weight-bearing time after surgery was determined based on the specific circumstances of the patient.

### Follow-up procedures

All patients received standardized outpatient follow-up at 1, 3, 6, and 12 months postoperatively. All clinical outcomes were evaluated by an independent surgeon who was not involved in the surgery and was unaware of the grouping. At 1-month follow-up, clinical assessments included wound healing status, pain evaluation using a visual analog scale, and initial ambulation ability. The anteroposterior and axial hip radiographs were obtained to assess early fracture alignment and prosthesis position. At 3-month follow-up, clinical evaluations focused on fracture healing progress assessed via tenderness mobility of fracture site, and abductor flexor muscle strength. The anteroposterior and axial hip radiographs were also obtained. Engh's score was calculated based on these images defined as low if <0 and high if ≥0. At 6-month follow-up, clinical assessments included continued fracture healing monitoring, range of motion of the hip joint, and complications such as heterotopic ossification and dislocation. The anteroposterior and axial hip radiographs were obtained to evaluate stem stability and bone integration. At 12-month final follow-up, comprehensive evaluations were performed, including confirmation of fracture healing, functional recovery assessment via Harris Hip Score, and final complications check. The anteroposterior and axial hip radiographs were reviewed to document long-term prosthesis position and bone ingrowth.

Each patient underwent a total of 8 anteroposterior and axial hip radiographs at each of the 4 follow-up time points. Engh's score was specifically obtained at 3 months while Harris Hip Scores were measured at the 12-month follow-up. The Harris Hip Score was exclusively measured at the 12-month because functional recovery after hip arthroplasty for intertrochanteric fractures typically requires sufficient time for fracture healing, soft tissue adaptation, and prosthesis stabilization. By 12-month postoperatively, fracture union is generally achieved, and the hip joint function reaches a relatively stable state, allowing for a reliable assessment of long-term functional outcomes. Earlier follow-up time points focus more on acute recovery processes such as wound healing, early fracture alignment, and initial complication screening, during which functional status remains dynamic and less representative of the final therapeutic effect. Thus, measuring the Harris Hip Score at 12 months ensures a valid and meaningful evaluation of the ultimate functional recovery.

### Radiographic evaluation protocol

**Figure 1:**
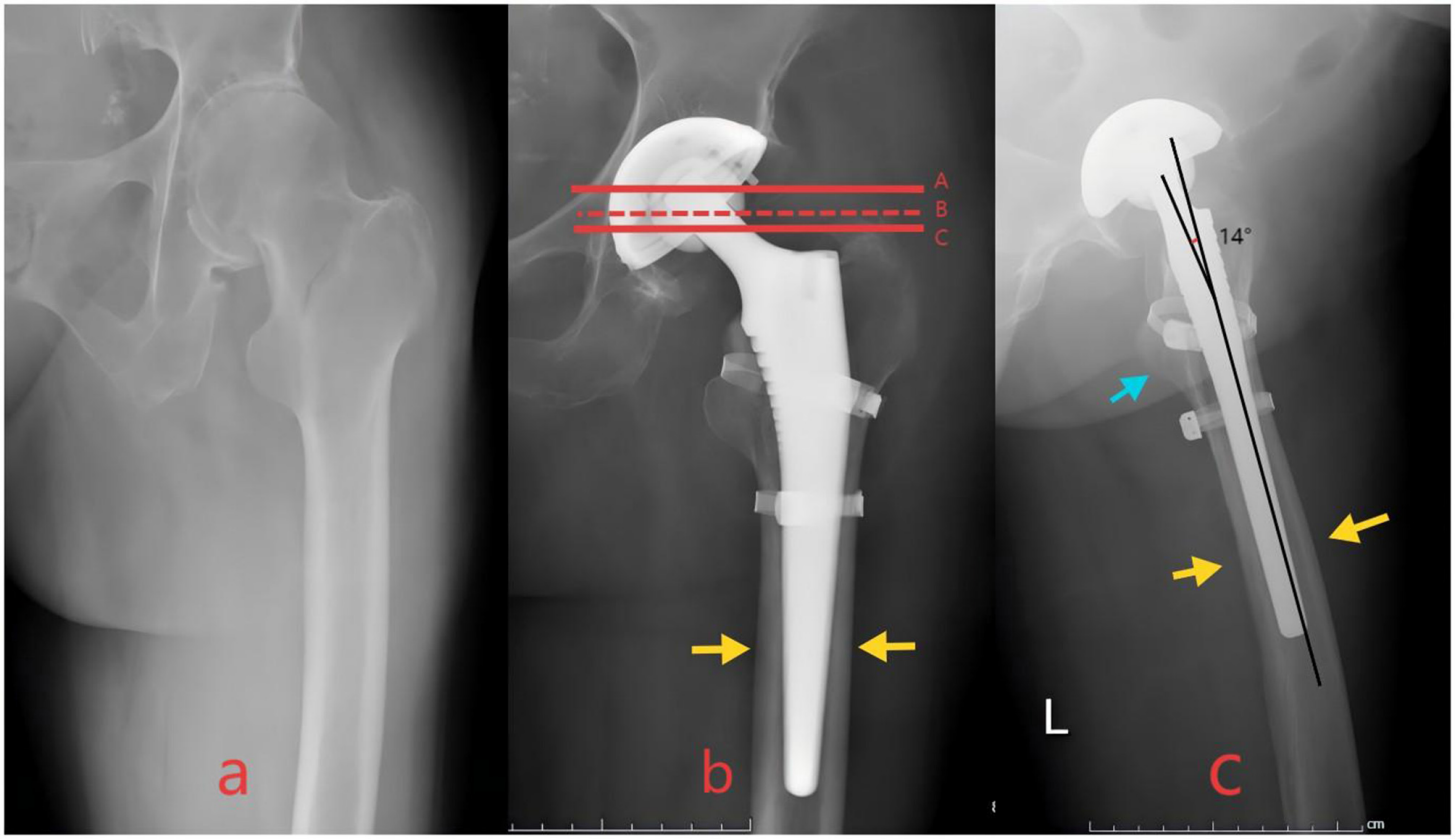
**(a)** Preoperative radiographs of a patient with intertrochanteric fracture. **(b**, **c)** Postoperative radiographs of a patient fulfilling all four predefined criteria for optimal alignment were classified as “Optimal Reduction.” Solid red lines A and C divided the prosthetic femoral head into three equal parts. Red dashed line B passed through the apex of the greater trochanter and between lines A and C. Blue arrows indicated no significant displacement of the lesser trochanter on the lateral view. Yellow arrows denoted optimal stem-canal fit with no radiolucent lines and cortical contact ≥80% on both views. The angle formed by two black lines, representing the intersection of the longitudinal axis of the femoral shaft and the longitudinal axis of the prosthetic femoral neck, was the femoral anteversion angle, measuring ~14°.

**Figure 2:**
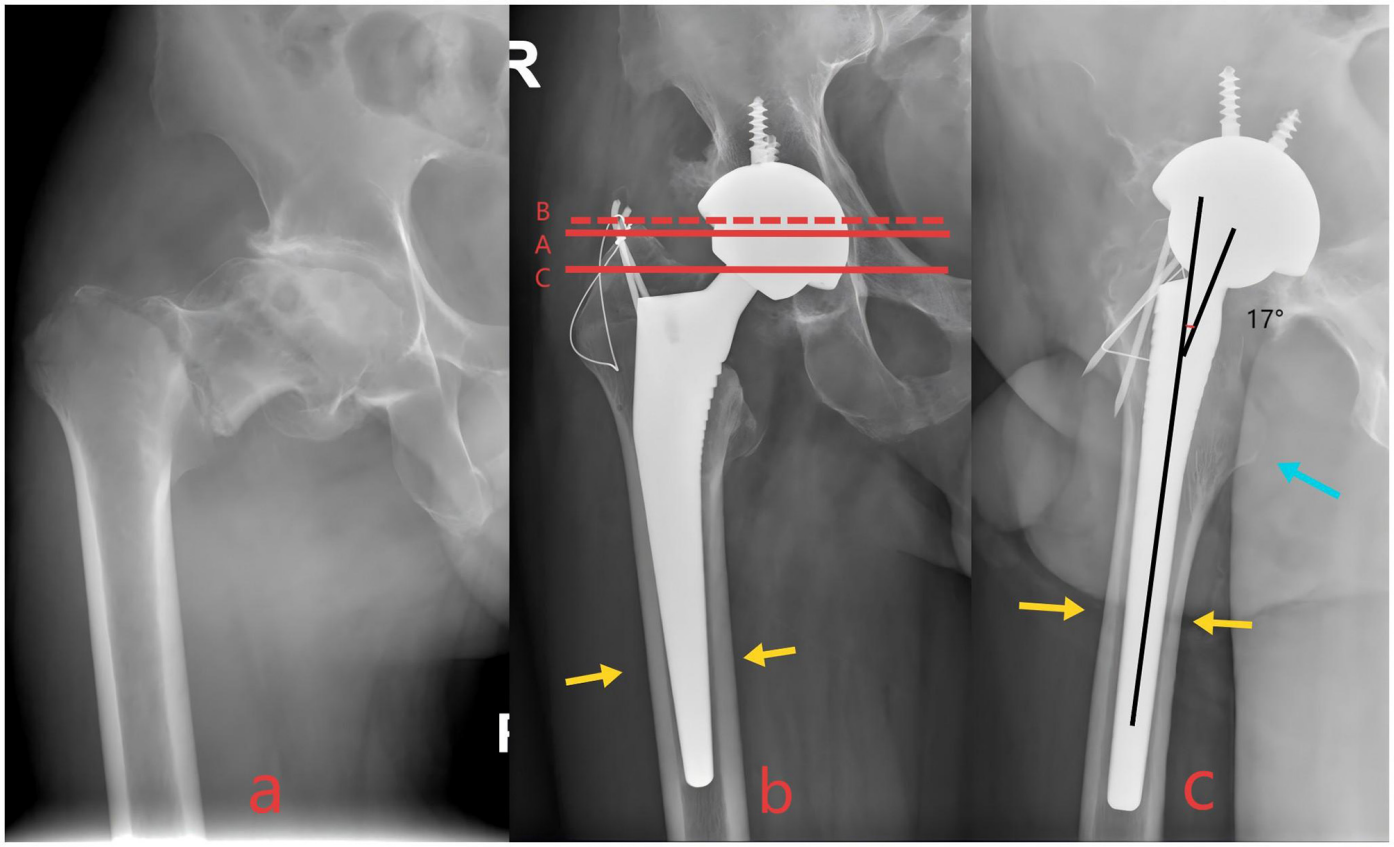
**(a)** Preoperative radiographs of a patient with intertrochanteric fracture. **(b**, **c)** Postoperative radiographs demonstrating acceptable alignment in two criteria, with none rated as poor, were categorized as “Acceptable Reduction.” Red dashed line B lied outside the area between lines A and C but remained within the superior and inferior margins of the prosthetic femoral head. Blue arrows indicated the lesser trochanter was well-positioned. Yellow arrows denoted optimal stem-canal fit with no radiolucent lines and cortical contact ≥80% on both views. Black lines represented the femoral anteversion angle, measuring ~17°.

**Figure 3:**
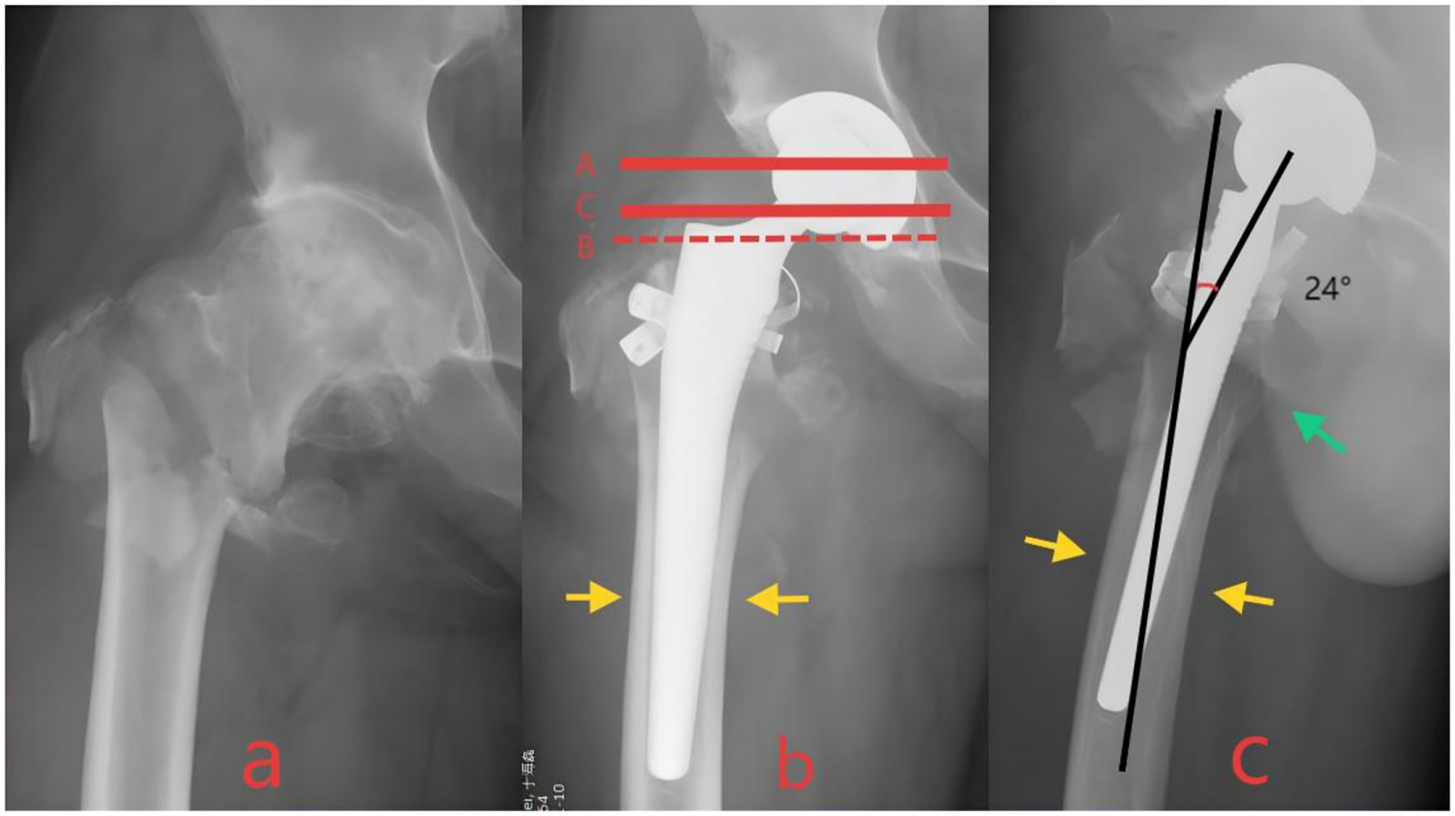
**(a)** Preoperative radiographs of a patient with intertrochanteric fracture. **(b**, **c)** Postoperative radiographs exhibiting at least one criterion of poor alignment were designated as “Poor Reduction”. Red dashed line B had extended beyond the inferior margin of the prosthetic femoral head. Blue arrows indicated poor reduction of the lesser trochanter. Yellow arrows denoted optimal stem-canal fit with no radiolucent lines and cortical contact ≥80% on both views. Black lines represented the femoral anteversion angle, measuring~24°.

The anteroposterior and axial radiographs of hip joints of these patients were collected after surgery. Yoon et al. (33) evaluated the reduction quality of open reduction and internal fixation for intertrochanteric fractures. Therefore, the reduction quality evaluation plan for hip arthroplasty for intertrochanteric fractures was based on their research reference. The quality of fracture reduction is evaluated by the following four criteria: (1) Greater trochanter alignment, defined as the apex positioning within the middle third of the prosthetic femoral head (optimal), upper/lower third without abductor laxity (acceptable), or displacement >5 mm (poor); (2) Lesser trochanter reduction, categorized by anatomic continuity with the medial cortex (optimal), ≤ 5 mm displacement (acceptable), or >5 mm avulsion (poor); (3) Femoral stem stability, evaluated by stem-canal fit (≥80% cortical contact, no radiolucency for optimal; 50%−80% contact with <5° angulation for acceptable; <50% contact or ≥5° malalignment for poor); and (4) Postoperative femoral anteversion angles were categorized as optimal (13 ± 3°), acceptable (6–10° or 16–20°), or poor (<6° or >20°) to optimize joint stability and reduce dislocation risk (Table 1). Compared to computed tomography, measuring femoral anteversion angle using axial femoral radiographs is a simpler, more economical method that reduces postoperative discomfort for patients. Its reliability has been validated by multiple studies (34–37). The specific measurement technique involved the following steps: on axial hip radiographs, the longitudinal axes of the femoral shaft and femoral neck were drawn, and the angle formed by the intersection of these two lines was defined as the femoral anteversion angle. These criteria prioritized biomechanical stability over anatomic perfection, reflecting the unique demands of arthroplasty in fracture management (Figures 1–3).

**Table 1:** Quality assessment of reduction of intertrochanteric fractures.

**Reduction quality**	**Optimal reduction**	**Acceptable reduction**	**Poor reduction**
Greater trochanter reduction	The apex of the greater trochanter aligns with the center of the prosthetic femoral head (middle third on anteroposterior view)	The apex lies within the upper or lower third of the prosthetic head	Displacement exceeding the upper/lower third of the prosthetic head or comminution
Lesser trochanter reduction	Anatomic alignment with the medial femoral cortex (no posterior displacement on lateral view)	Mild displacement (≤ 5 mm) without compromising iliopsoas attachment	Displacement >5 mm or complete avulsion, leading to iliopsoas dysfunction
Femoral stem stability	Optimal stem-canal fit with no radiolucent lines and cortical contact ≥80% on both views	Minor stem malalignment (<5° angulation) with 50%−80% cortical contact	Significant malalignment (≥5° angulation), subsidence, or cortical contact <50%
Femoral anteversion restoration	Femoral anteversion angle 13 ± 3°	Femoral anteversion angle 6–10°or 16–20°	Femoral anteversion angle <6°or >20°

To ensure evaluation accuracy, these radiographs were independently reviewed in a blinded manner by two orthopedic surgeons who were unaware of patient group assignments and the study objectives; any discrepancies were resolved by a third orthopedic surgeon. To minimize measurement error and assess reliability, all radiographic measurements were repeated by one of the researchers after a 6-week interval. Intraclass correlation coefficients (ICCs) were calculated to evaluate both intra-observer and inter-observer reliability. An ICC value ≥ 0.8 was considered indicative of good reliability, and ≥ 0.9 indicated excellent reliability.

### Reduction quality grading system

The reduction quality was stratified into three grades based on integrated radiographic criteria: (1) Optimal reduction (Grade A): all radiographic parameters achieved optimal status (Figure 1). (2) Acceptable reduction (Grade B): up to two radiographic parameters were classified as acceptable (Figure 2). (3) Poor reduction (Grade C): any radiographic parameter categorized as poor (Figure 3). Fracture reduction quality grade of each case was determined according to the criteria. Based on the quality grade of fracture reduction, the follow-up information of cases in three grades was statistically analyzed. In order to prevent the impact of fracture classification, we calculated adjusted *P-values* based on fracture classification (AO/OTA).

### Ethical approval

This retrospective study was approved by the Ethics Committee of Hebei Medical University Third Hospital. Informed consent was waived due to the anonymized and retrospective nature of the data. All procedures complied with the ethical standards of the Declaration of Helsinki and relevant national regulations to ensure patient privacy and data security.

### Statistical analysis

All statistical analyses were performed using SPSS 26.0 software. Continuous variables were expressed as mean ± standard deviation, and categorical variables were presented as frequencies. For comparisons of continuous variables among the three groups, one-way analysis of variance was applied. *Post-hoc* pairwise comparisons were conducted using Tukey's honestly significant difference test to control for Type I error associated with multiple comparisons. For categorical variables, chi-square test was used, or Fisher's exact test was employed when expected cell counts were <5. To eliminate potential confounding by fracture complexity, all statistical results were adjusted for AO/OTA fracture classification using analysis of covariance for continuous outcomes and logistic regression for categorical outcomes. The adjusted *P-values* were reported to reflect these corrections. An a priori power analysis using G-Power software version 3.1.9.7 (Heinrich-Heine-Universitat Dusseldorf, Dusseldorf, Germany) showed that 53 patients in each group could detect significant difference at 80% power.

## Results

### Patient demographics and baseline characteristic

**Figure 4:**
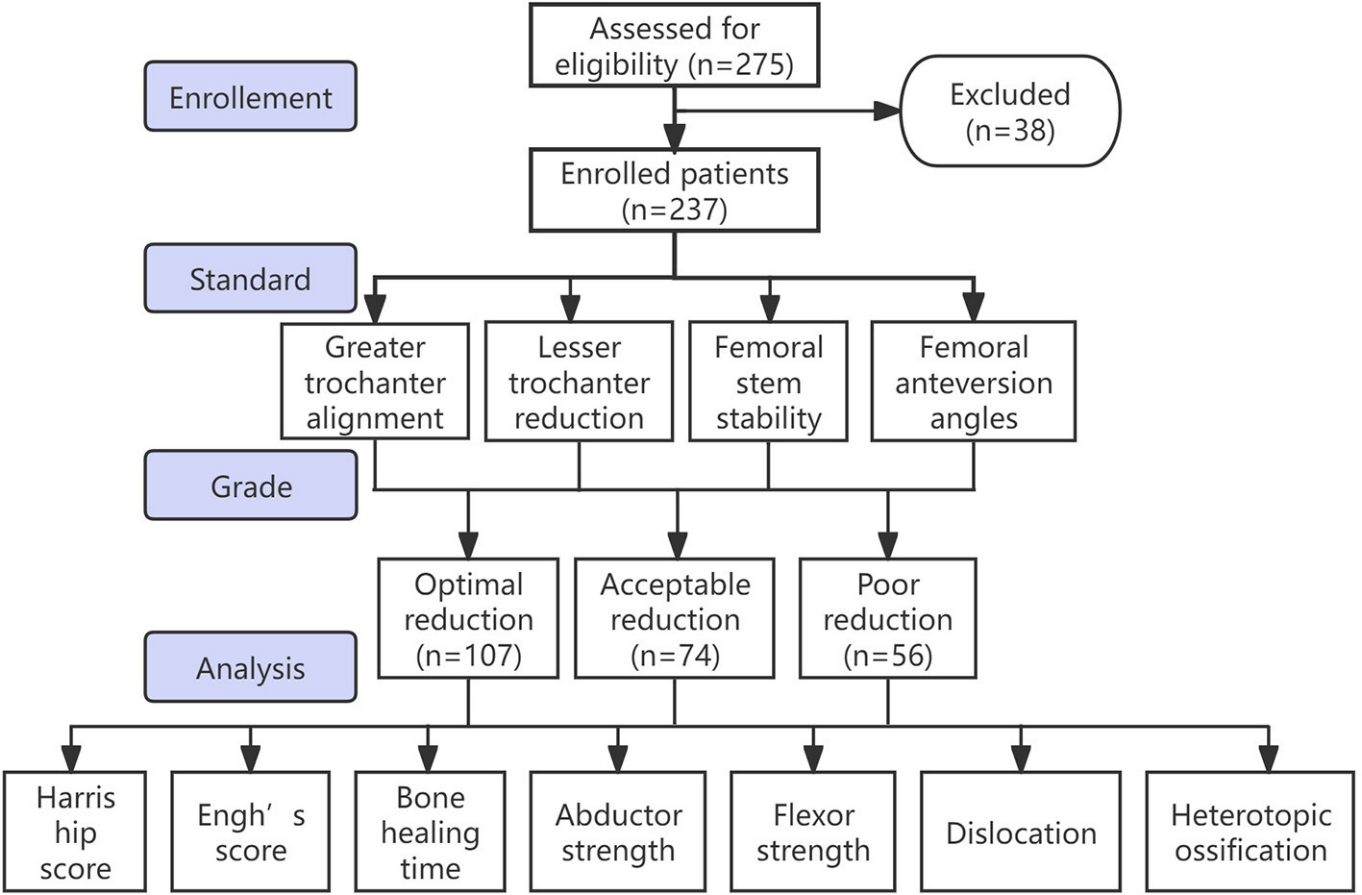
Flow diagram of the research. According to the quality of reduction, patients were divided into three groups: perfect reduction, acceptable reduction, and poor reduction.

In the end, 99 male (41.77%) and 138 female (58.23%) patients were included in this study. Their demographic characteristics were shown in Table 2. According to the quality of reduction, patients were divided into three groups: perfect reduction (*n* = 107, 45.15%), acceptable reduction (*n* = 74, 31.22%) and poor reduction (*n* = 56, 23.63%; Figure 4). The average follow-up time for the three groups was 4.65 ± 2.39 years, 4.95 ± 2.37 years, and 4.96 ± 2.11 year, respectively. There were no significant differences in age, gender, body mass index, and bone mineral density among the three groups of patients (*P* > 0.05).

**Table 2:** Baseline characteristics of patients with intertrochanteric fractures undergoing hip arthroplasty.

**Baseline characteristics**	**Perfect reduction (*n* = 107)**	**Acceptable reduction (*n* = 74)**	**Poor reduction (*n* = 56)**	***P***
Age (years)	70.41 ± 10.28	71.50 ± 9.70	70.46 ± 8.46	0.733
Sex (Male/Female)	41/66	33/41	25/31	0.623
Body mass index (kg/m^2^)	24.22 ± 3.26	24.03 ± 3.11	23.87 ± 2.71	0.782
**Comorbidity**				
Hypertension	43	24	16	0.290
Diabetes	22	13	11	0.882
Cardiovascular disorders	17	13	13	0.512
Neurological disorders	24	15	10	0.789
Classification of intertrochanteric fracture				<0.001
31–A1	61	20	10	
31–A2	34	45	38	
31–A3	12	9	8	
Bone mineral density (g/cm3)	−2.28 ± 1.15	−2.38 ± 1.22	−2.26 ± 1.17	0.802
Follow-up time (years)	4.65 ± 2.39	4.95 ± 2.37	4.96 ± 2.11	0.595

### Surgical procedural details

There was no statistically significant difference in surgical time and intraoperative bleeding among the three groups of patients. In the selection of femoral stems during surgery, the group with perfect reduction mostly used type IV femoral stems, with a small number of type V femoral stems and a very small number of type III femoral stems (79 vs. 23 vs. 5). The acceptable reduction group consisted mainly of type IV and V femoral stems, with a small amount of type III femoral stems (46 vs. 25 vs. 3). The poor reduction group was mainly composed of type IV femoral stems (39 vs. 15 vs. 2). In the selection of intraoperative internal fixation devices, both cable and tension were the most commonly used internal fixation devices in the three groups (Table 3).

**Table 3:** Characteristics of surgical process of patients with intertrochanteric fractures undergoing hip arthroplasty.

**Characteristics**	**Perfect reduction (*n* = 107)**	**Acceptable reduction (*n* = 74)**	**Poor reduction (*n* = 56)**	***P***
Operative time (min)	128.74 ± 32.54	125.14 ± 36.78	130.09 ± 22.53	0.641
Bleeding volume (ml)	434.11.21 ± 166.77	378.38 ± 134.24	409.82 ± 141.56	0.053
Femoral stem type				0.422
Type III	5	3	2	
Type IV	79	46	39	
Type V	23	25	15	
Internal fixation				0.316
Nothing	11	7	8	
Cable only	51	37	30	
Tension band	27	17	9	
Plate with screws or cables	3	9	0	
Others	15	11	9	
Postoperative weight-bearing time				<0.001
Immediately	80	56	39	
≤ 4-week non-weight-bearing time	26	15	14	
> 4-week non-weight-bearing time	1	3	3	

### Fracture healing and radiographic integration

The ICC values indicated good to excellent intra-observer and inter-observer reliability for all measurements, with the intra-observer ICC values ranging from 0.878 to 0.941 and inter-observer ICC values ranging from 0.861 to 0.929. In the perfect reduction group, 4 patients (3.74%) were diagnosed with delayed fracture healing, while in the acceptable reduction group, there were 8 patients (10.81%). In the group with poor reduction quality, 8 patients (14.29%) were diagnosed with delayed fracture healing (*P* = 0.031). Among the three groups, the proportion of high Engh's scores was 97.20%, 82.43%, and 73.21%, respectively (*P* = 0.002).

### Functional outcomes

The average Harris score of the hip joint in the perfect reduction group was 92.57 ± 4.27 (77–99). The Harris score of the hip joint in the group with acceptable reduction quality was relatively low, which is 84.35 ± 4.89 (78–93). The Harris score of the hip joint in the group with poor reduction quality was 82.46 ± 7.05 (52–91), which was the lowest among the three groups (Table 4).

**Table 4:** Clinical outcomes of hip arthroplasty for intertrochanteric fractures.

**Clinical outcomes**	**Perfect reduction (*n* = 107)**	**Acceptable reduction (*n* = 74)**	**Poor reduction (*n* = 56)**	${\text{P}}_{1}^{*}$	** ${\text{P}}_{2}^{\text{\#}}$ **
Harris hip score	92.57 ± 4.27	84.35 ± 4.89	82.46 ± 7.05	<0.001	<0.001
Harris hip score excellent/poor ratio				<0.001	<0.001
Excellent (80–100)	105	61	39		
Poor (<80)	2	13	17		
Engh's score				<0.001	0.002
Unstable (<−10)	0	0	3		
Suboptimum, but Stable (−10 to 0)	3	13	12		
Ingrowth suspected (0–10)	46	29	20		
Bone Ingrown (>10)	58	32	21		

^*^P_1_ represented unadjusted statistical significance values from initial analyses.

^#^P_2_ represented adjusted P-values after correcting for AO/OTA fracture classification, with the correction based on this classification. For continuous outcomes, analysis of covariance was applied; for categorical outcomes, logistic regression with AO/OTA classification as a covariate was used.

### Muscle strength assessment

After evaluation, a total of 2 patients were diagnosed with abductor weakness in the perfect reduction group (1.87%). In the acceptable reduction group, 7 patients were diagnosed with abductor weakness (9.46%). In the group with poor reduction quality, 8 patients diagnosed with abductor weakness increased this proportion to 14.29% (*P* = 0.014). Meanwhile, the patient's hip flexor muscle strength was measured. When the flexor muscle strength was between 4–5 levels, it is considered normal flexor strength, while flexor weakness is considered muscle strength less than or equal to 3 levels. Only one patient was diagnosed with flexor weakness in the perfect reduction group (0.93%). However, in the acceptable reduction group and the poor reduction group, this number increased to 5 cases (6.78%) and 6 cases (10.71%), respectively (*P* = 0.020; Table 5).

**Table 5:** Postoperative complications of hip arthroplasty for intertrochanteric fractures.

**Postoperative complications**	**Perfect reduction (*n* = 107)**	**Acceptable reduction (*n* = 74)**	**Poor reduction (*n* = 56)**	${\text{P}}_{1}^{*}$	** ${\text{P}}_{2}^{\text{\#}}$ **
**Bone healing time**				0.048	0.031
≤ 3 months	103	66	48		
>3 months	4	8	8		
**Abductor strength**				0.009	0.014
≤ 3	2	7	8		
4–5	105	67	48		
**Flexor strength**				0.018	0.020
≤ 3	1	5	6		
4–5	106	69	50		
**Dislocation**				0.195	0.278
Yes	2	2	4		
No	105	72	52		
**Heterotopic ossification**				0.833	0.897
Yes	5	3	3		
No	102	71	53		

^*^P_1_ represented unadjusted statistical significance values from initial analyses.

^#^P_2_ represented adjusted P-values after correcting for AO/OTA fracture classification, with the correction based on this classification. For continuous outcomes, analysis of covariance was applied; for categorical outcomes, logistic regression with AO/OTA classification as a covariate was used.

### Complications

The number of cases of hip dislocation in the three groups of patients is 2, 4, and 4, respectively (1.87% vs. 2.70% vs. 7.14%), but there was no statistical significance (*P* = 0.278). The number of cases of heterotopic ossification in the three groups was 5, 3, and 3, respectively (4.67% vs. 4.05% vs. 5.36%), and there was no statistical significance (*P* = 0.897; Table 5).

## Discussion

Our findings further validated the rationale for hip arthroplasty as a preferred treatment for unstable intertrochanteric fractures, particularly in elderly patients with osteoporosis or hip joint diseases such as osteoarthritis or femoral head necrosis. Hip arthroplasty addresses these limitations by providing immediate structural stability and reducing the risks associated with fixation failure. However, the success of arthroplasty hinges significantly on the quality of fracture reduction and fixation, as demonstrated in our study. This underscores the need for refinement in surgical techniques and evaluation standards to further bridge the gap between the advantages of arthroplasty and its dependence on reduction quality.

There has been controversy over how to reduce fracture fragments and evaluate the quality of reduction during hip arthroplasty. Lee et al. (30) used the Ethibond Suture technique to firmly fix the greater trochanter of the fracture during hip arthroplasty, and found that good fixation of the greater trochanter can lead to better prognosis for patients. However, unlike previous studies, we quantified the impact of lesser trochanter reduction on hip flexion strength, highlighting its biomechanical role in load distribution. Zhang et al. (38) found through 2–11 years of follow-up that the use of Kirschner wires and tension band in hip arthroplasty can achieve good therapeutic effects for intertrochanteric fractures. Grimsrud et al. (39) treated unstable intertrochanteric fractures using a novel cerclage cable technique during surgery. There have been lots of studies (40–42) on the quality of fracture reduction in intertrochanteric fractures, but most of these studies have focused on internal fixation rather than hip arthroplasty.

The fracture reduction quality was divided into three levels and the differences in therapeutic effects caused by different reduction qualities were evaluated. The results demonstrated that better reduction quality significantly correlated with improved clinical outcomes, as evidenced by higher Harris hip scores and Engh's scores, faster fracture healing, and lower rates of complications such as abductor and flexor muscle weakness. These findings aligned with existing literature emphasizing the critical role of anatomical reduction in promoting optimal load distribution, mechanical stability, and effective osseointegration of the prosthesis. Optimal reduction directly correlated with restored hip biomechanics. Grade A patients demonstrated near-normal abductor strength and flexion capacity, whereas Grade C reductions were associated with functional deficits. This aligned with biomechanical models highlighting the dependence of hip kinematics on trochanteric integrity and stem stability. Notably, even minor malreductions (Grade B) led to measurable functional declines, underscoring the need for stringent reduction criteria.

Reduction and fixation of the greater and lesser trochanters were particularly significant for postoperative function. Proper fixation of the greater trochanter supported better abductor muscle recovery, reducing the risk of gait abnormalities and weakness. Similarly, the fixation of the lesser trochanter proved essential for preserving hip flexion strength. These findings corroborated earlier studies on the biomechanical relevance of trochanteric stabilization in hip arthroplasty (43). Similarly, the lesser trochanter, essential for hip flexion, and external rotation, must be adequately reduced and stabilized to prevent postoperative flexor weakness. Inadequate fixation of these regions, as seen in patients with poor reduction quality, led to weaker hip flexor and abductor muscle strength in our study. Patients with Grade A reductions exhibited superior Harris Hip Scores and lower rates of delayed union, emphasizing the biomechanical advantages of precise reduction in arthroplasty. Dislocation rates, though statistically insignificant across groups, trended higher in Grade C, likely due to abductor insufficiency and altered joint mechanics. These results advocated for prioritizing greater trochanter fixation to mitigate instability. These findings suggested that meticulous intraoperative reduction is not merely a technical preference but a prognostic imperative, particularly in osteoporotic patients prone to instability. Our findings aligned with earlier studies, such as those by Lee et al. (30) and Zhang et al. (38), which reported that techniques like Ethibond suture fixation or Kirschner wire application significantly improved postoperative outcomes by enhancing trochanteric stability.

The anatomical reduction of the posteromedial cortex is the most important component in the reduction of intertrochanteric fractures (33). However, these elderly patients often suffer from osteoporosis or comminuted fractures, greatly increasing the difficulty of reducing the posteromedial cortex (42, 44). Patients with cortical contact <50% (Grade C) exhibited higher instability rate evaluated by Engh's score, reflecting poor osseointegration. This corroborated the study of Haidukewych et al. (41) emphasizing the cortical support for load distribution, suggesting that even imperfect reductions must prioritize cortical continuity to prevent stem subsidence. Our study emphasizes that the anterior medial cortex, although less challenging to visualize and align, plays a crucial role in supporting the femoral prosthesis stem, as its integrity significantly impacts femoral stem stability and bone ingrowth. Prior research supported this focus on achieving cortical continuity and stability (43).

Haidukewych et al. (41) reported that hip inversion or hip eversion can be evaluated by the positional relationship between the greater trochanter and the femoral head, and the best effect can be achieved when the horizontal line where the apex of the greater trochanter is located passes through the center of the femoral head. Considering that the recovery of femoral anteversion and neck-shaft angle is extremely important in both hip arthroplasty and reduction of intertrochanteric fractures, we applied the research of Haidukewych et al. to hip arthroplasty and achieved good results (41).

Beyond functional scores, our study observed a notable correlation between reduction quality and fracture healing. Delayed healing was significantly more common in patients with poor reduction quality, emphasizing the importance of anatomical alignment for promoting osteogenesis and reducing complications. Delayed union rates escalated with poorer reductions, likely due to disrupted vascularity and mechanical instability. Notably, delayed healing itself exacerbated functional deficits, as seen in lower Harris scores. These findings mirrored Huang et al.'s (15) meta-analysis linking anatomic reduction to accelerated union, highlighting a bidirectional relationship: reduction quality drives healing, while healing underpins functional recovery.

Prosthesis selection in hip arthroplasty for intertrochanteric fractures is critical, with options varying by design, fixation, and indication. Cemented calcar-replacement and long-stem cemented hemiarthroplasty showed similar clinical outcomes in octogenarians with unstable fractures (45). Bipolar hemiarthroplasty is preferable over total hip arthroplasty in elderly osteoporotic patients due to shorter operative time, less blood loss, and lower dislocation rates (46). Cementless long stems with double cross binding technique achieved good mid-term results in octogenarians, with stable fixation and successful trochanteric healing (47). For failed fixation, cementless revision stems were safer in early failures to reduce reoperations, while primary stems worked in late failures (48), and primary cementless stems yielded comparable outcomes to revision stems in conversion arthroplasty with shorter hospital stays (49). However, for the independence of implant type selection from reduction quality, due to the small sample size and large variety of prostheses, we were unable to accurately assess this relationship, which should be further studied in the large-scale research.

This study's findings validated the reduction quality evaluation method adapted from Yoon et al. (33), demonstrating its utility in postoperative assessments. While intramedullary nailing remains first-line for most intertrochanteric fractures, hip arthroplasty offers immediate stability in select cases where anatomical reduction is unachievable or pre-existing joint degeneration warrants definitive management (17, 25). Our findings support hip arthroplasty as a viable option for elderly patients with complex fractures or osteoporosis, prioritizing early mobilization to mitigate systemic risk. Further research should explore the long-term durability of outcomes across varying reduction qualities and examine potential advancements in fixation techniques, especially for complex fracture patterns. In conclusion, achieving high-quality reduction during hip arthroplasty for intertrochanteric fractures is paramount for optimizing functional recovery and minimizing complications. Our findings advocate for meticulous preoperative planning and intraoperative strategies to enhance reduction quality, thereby improving patient prognosis and quality of life postoperatively.

This study has several limitations that should be considered when interpreting the results. First, while postoperative radiographs were reviewed independently by two orthopedic surgeons in a blinded manner, we did not explicitly address masking protocols for outcome assessors, quantify inter-rater reliability for reduction quality grading, or systematically document the independence of outcome assessors, which may introduce potential biases and uncertainties in the consistency and objectivity of assessments. Second, as a single-center retrospective study with a sample restricted to patients aged 55–90 years meeting specific arthroplasty indications, the findings lack sufficient discussion of external validity and generalizability, limiting their applicability to other populations or institutions with varying surgical practices. Third, the measurement of femoral anteversion relied on axial radiographs, which, though validated, are less precise than computed tomography, potentially introducing minor inaccuracies in anteversion classification and affecting reduction quality stratification. Additionally, due to the relatively small sample size and the wide variety of hip prostheses used, we were unable to analyze the relationship between prosthesis type and reduction quality, leaving unresolved questions about potential interactions between implant selection and reduction outcomes.

## Conclusion

This study introduced and validated a standardized radiographic evaluation system to assess reduction quality in arthroplasty for intertrochanteric fractures, emphasizing the prognostic importance of anatomic trochanteric alignment and cortical continuity. High-quality reduction is critical for optimizing functional recovery and minimizing complications in arthroplasty for intertrochanteric fractures. Future research should explore long-term outcomes and advanced fixation techniques to enhance reduction precision.

## Data Availability

The raw data supporting the conclusions of this article will be made available by the authors, without undue reservation.
